# Sea-Level Estimation from GNSS-IR under Loose Constraints Based on Local Mean Decomposition

**DOI:** 10.3390/s23146540

**Published:** 2023-07-20

**Authors:** Zhenkui Wei, Chao Ren, Xingyong Liang, Yueji Liang, Anchao Yin, Jieyu Liang, Weiting Yue

**Affiliations:** College of Geomatics and Geoinformation, Guilin University of Technology, Guilin 541004, China3222080807217@glut.edu.cn (X.L.); lyjayq@glut.edu.cn (Y.L.); yinanchao@glut.edu.cn (A.Y.); liangjieyu@glut.edu.cn (J.L.);

**Keywords:** GNSS-IR, SNR, sea-level estimation, LMD, loose constraints

## Abstract

The global navigation satellite system–interferometric reflectometry (GNSS-IR) technique has emerged as an effective coastal sea-level monitoring solution. However, the accuracy and stability of GNSS-IR sea-level estimation based on quadratic fitting are limited by the retrieval range of reflector height (RH range) and satellite-elevation range, reducing the flexibility of this technology. This study introduces a new GNSS-IR sea-level estimation model that combines local mean decomposition (LMD) and Lomb–Scargle periodogram (LSP). LMD can decompose the signal-to-noise ratio (SNR) arc into a series of signal components with different frequencies. The signal components containing information from the sea surface are selected to construct the oscillation term, and its frequency is extracted by LSP. To this end, observational data from SC02 sites in the United States are used to evaluate the accuracy level of the model. Then, the performance of LMD and the influence of noise on retrieval results are analyzed from two aspects: RH ranges and satellite-elevation ranges. Finally, the sea-level variation for one consecutive year is estimated to verify the stability of the model in long-term monitoring. The results show that the oscillation term obtained by LMD has a lower noise level than other signal separation methods, effectively improving the accuracy of retrieval results and avoiding abnormal values. Moreover, it still performs well under loose constraints (a wide RH range and a high-elevation range). In one consecutive year of retrieval results, the new model based on LMD has a significant improvement effect over quadratic fitting, and the root mean square error and mean absolute error of retrieval results obtained in each month on average are improved by 8.34% and 8.87%, respectively.

## 1. Introduction

Glaciers are melting in large quantities as global warming intensifies, leading to sea-level rise and a constant threat to human life [1]. Consequently, real-time tracking of sea-level variations and studying related patterns is of paramount importance [2]. Over the past two decades, traditional tide gauges (TG) have been used as the primary means of sea-level monitoring. However, their measured results are subject to error due to the influence of crustal movements. Although satellite altimetry can achieve high-accuracy and large-scale sea-level monitoring, its monitoring accuracy is low in the near-coastal area [3]. In recent years, with the maturity of global positioning system–interferometric reflectometry (GNSS-IR) technology, the sea-level estimation method based on this technology provides an effective solution for near-coastal sea-level monitoring. GNSS-IR technology enables the cost-effective, uninterrupted monitoring of sea levels, and the resulting observations are automatically anchored in a stable frame [4].

In 1993, Martin-Neira, a scientist at the European Space Agency (ESA), initially introduced global navigation satellite system–reflectometry (GNSS-R) technology, which has become one of the focuses of research in the field of remote sensing [5]. This technique uses the characteristic parameters of the reflected signal to detect the physical characteristics of the reflecting surface and has a high application value [6,7,8,9]. In 2008, Larson et al. further proposed the GNSS-IR technique and successfully retrieved the variation of soil moisture [10,11]. The technique estimates relevant parameters of the reflecting surface through the characteristics of signal-to-noise ratio (SNR) oscillation (such as frequency, amplitude, and initial phase). With the advantages of low cost, high flexibility, and accessible data acquisition, GNSS-IR technology has been applied in many fields, such as snow depth detection [12,13], soil moisture [14,15], storm surge [16,17], and vegetation change [18,19].

In 2013, Larson et al. systematically described the general method of retrieving sea-level height using GNSS-IR technology, therefore establishing the foundation of GNSS-IR sea-level measurement technology [20]. Lofgren et al. used SNR and phase delay analysis to retrieve the water level variation in the rough sea surface, revealing the superior performance of the SNR analysis method [21]. Given the irrationality of the static sea-level assumption in the retrieval principle, Larson et al. proposed a dynamic sea-level correction method. It was applied to Kachemak Bay, where the tidal level variation was greater than 7 m, and the retrieval results were obtained with the root mean square error (RMSE) of 2.3 cm [22]. Williams et al. found that tropospheric delay could bias sea-level estimates. To address this problem, they proposed using the Global Temperature and Pressure (GPT2w) and Vienna mapping functions (VMF1) to eliminate the effect of tropospheric delay on sea-level retrieval [23]. Jia et al. first used the SNR data of L2, L6, and L7 bands in the BeiDou system to retrieve sea-level height and validated its availability in the GNSS-IR technology for sea-level estimation [24]. Wang et al. analyzed the SNR data from three sites, PBAY, SC02, and BRST, determining the optimal azimuth range for sea-level monitoring at each site [25]. Wang et al. used wavelet analysis to process SNR arc and extract instantaneous frequencies, significantly improving the data utilization rate [26]. Wang et al. used a robust regression method to combine SNR data from quad constellations of GNSS to retrieve the sea-level height, and the accuracy was improved by about 40–75% [27]. After years of development, GNSS-IR sea-level measurement technology has matured significantly. In this technique, the accuracy of the retrieval result largely depends on the quality of the oscillatory term [28]. The traditional model uses quadratic fitting to separate the trend and oscillation terms of the SNR arc. However, this method offers inadequate signal separation and introduces complex noise in the oscillation term, which is not conducive to extracting oscillation frequency [29,30]. Although the effect of noise can be weakened to some extent by setting a retrieval range for the reflector height (RH range) and using the SNR series with a low-elevation range, it also reduces the flexibility of the technique. To this end, Wang et al. used wavelet decomposition to process the SNR arc and selected signal components containing information about the sea surface to construct the oscillation term [31]. Zhang et al. and Hu et al. processed SNR arc using empirical modal decomposition (EMD) and variational modal decomposition (VMD), respectively, which effectively improved the accuracy and stability of retrieval results in the high-elevation range [32,33]. This refined model, based on signal processing, enhances retrieval accuracy by extracting the oscillation term with as low noise as possible from the SNR arc. In addition, the B-sample [34], wavelet decomposition [31], EMD [32], VMD [33], and singular spectrum analysis (SSA) [35] have all been applied to the GNSS-IR sea-level estimation model, all of which have improved the accuracy and stability of retrieval results to some extent. Nevertheless, it is still challenging for technology to achieve long-term, accurate, stable sea-level monitoring. In previous studies, the focus has been on exploring the advantages of improved methods based on signal decomposition at high satellite-elevation ranges, overlooking the impact of the RH range on retrieval accuracy. It is important to note that the elevation range is primarily influenced by the visual range of the antenna. As short-term and long-term tide-level fluctuations often exhibit different elevation ranges, it is essential to appropriately expand the RH range to capture the comprehensive sea-level elevation variation in long-term monitoring. Moreover, the mechanism behind the signal-decomposition method remains unclear in existing research.

To further enhance the retrieval accuracy and stability of the GNSS-IR sea-level estimation model, this study proposes a GNSS-IR model based on local mean decomposition (LMD) for sea-level estimation. The model uses LMD instead of quadratic fitting to process the SNR arc to obtain the oscillation term and uses the Lomb–Scargle periodogram (LSP) to extract the oscillation frequency. LMD can decompose the SNR arc into a series of signal components, and those that contain the sea surface information are selected to construct the oscillation term with a low noise level. Observations from the SC02 site are used to analyze the performance of LMD and the effect of noise on retrieval results from RH ranges and satellite-elevation ranges, and the stability of the model is verified by retrieving the sea-level variation at Friday Harbor for one year.

The rest of this paper is organized as follows. Section 2 introduces the experimental site and data. Section 3.1 introduces the basic principle of GNSS-IR technology, and Section 3.2 briefly introduces the decomposition principle of LMD. Section 4 describes the experimental procedure in detail. In Section 5, experimental results are given and discussed. Finally, Section 6 offers the conclusion and final remarks on the paper.

## 2. Site and Data

The SC02 site (48°32′46.30″ N, 123°00′27.40″ W) is located northeast of Friday Harbor, Washington, USA, and is one of the observation sites of the Plate Boundary Observatory (PBO) operated by UNAVCO for EarthScope. It is equipped with a geodetic receiver TRIMBLE NETR9 and choke antenna with rectifier TRM59800.80. The antenna is erected on the bedrock by the coast, its phase center is about 5.5 m from the sea level, and the azimuth range of 50°~240° is the sea surface [25,36]. The Friday Harbor TG, about 300 m west of SC02, provides 6-min tidal level monitoring data, which is operated by the National Oceanic and Atmospheric Administration (NOAA) of the United States.

**Figure 1:**
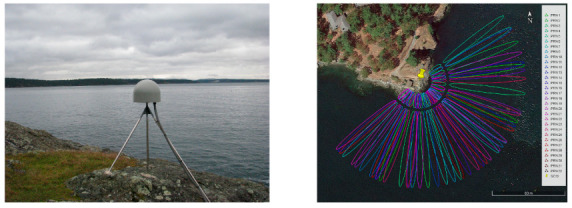
(**Left**) Surroundings of the SC02 site (http://www.unavco.org (accessed on 18 March 2023)). (**Right**) First Fresnel zone of the SC02 site. Fresnel reflection zones of 5° and 15° are plotted on the Google Earth image, with the yellow pin indicating site location and ellipses of different colors corresponding to reflection zones of different satellites.

This study used the SNR data of the Global Positioning System (GPS) L1 band at the SC02 site in 2015 with a sampling interval of 15 s, and the accuracy of retrieval results was evaluated using TG data at Friday Harbor. The first Fresnel zone (FFZ) is typically used to represent the sensing range of the signal on the reflecting surface. With a fixed RH, its position and size are determined by the azimuth and elevation of the satellite, respectively. As the elevation increases, the length of the reflector zone diminishes, and the center moves closer to the antenna. In this study, the SNR series in the elevation range of 0.5°~15° was used to retrieve the sea-level height, and the number of SNR observations should be greater than 100 in one observation period. The SNR series with elevations up to 35° was also used to explore the performance of the model at high elevations. Figure 1 shows the surrounding environment of the SC02 site and the FFZ with satellite elevations of 5° and 15°, and the radius of the reflection zone is about 100 m [37].

## 3. Methodology

### 3.1. GNSS Interferometric Reflection Theory

**Figure 2:**
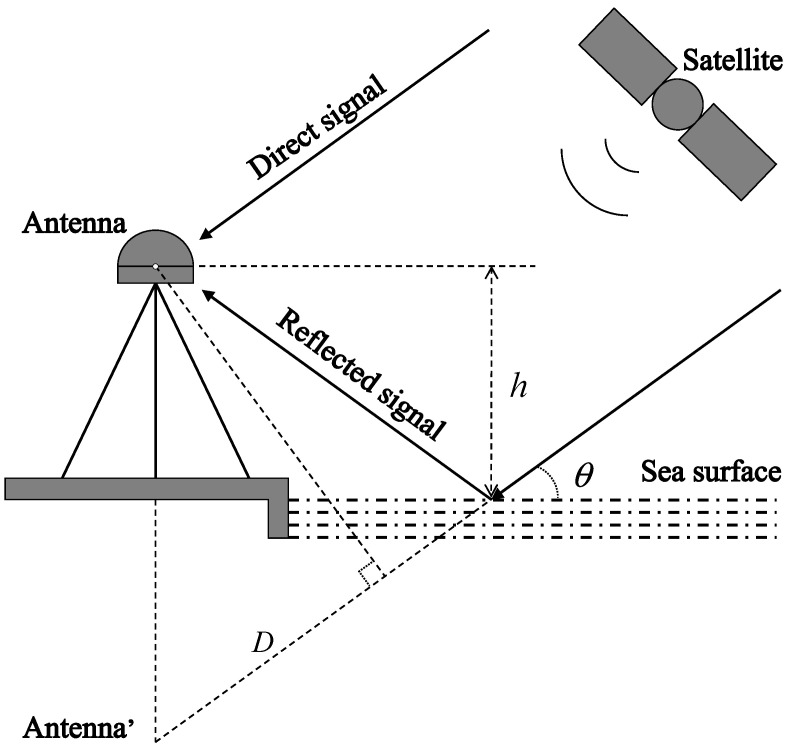
Schematic diagram of GNSS-IR geometry.

To maximize the reception of the reflected signals from the sea surface, antennas of GNSS receivers intended for sea-level estimation are typically installed near the coast. The signal received by the receiver can be divided into two categories: one is the direct signal received directly after the satellite launch, and the other is the reflected signal reflected by the sea surface and then enters the receiver. Figure 2 shows the geometry of the GNSS-IR model, where h is the vertical distance from the antenna phase center to the sea surface, i.e., the RH; θ is the angle between the direct signal and sea level, i.e., the satellite-elevation angle; and D is the additional distance of the reflected signal compared to the direct signal.

If only one reflection from a calm reflecting surface is considered, the additional distance D can be deduced from the geometric relationship as:

(1) $$D=2h\sin\left(\theta \right)$$

**Figure 3:**
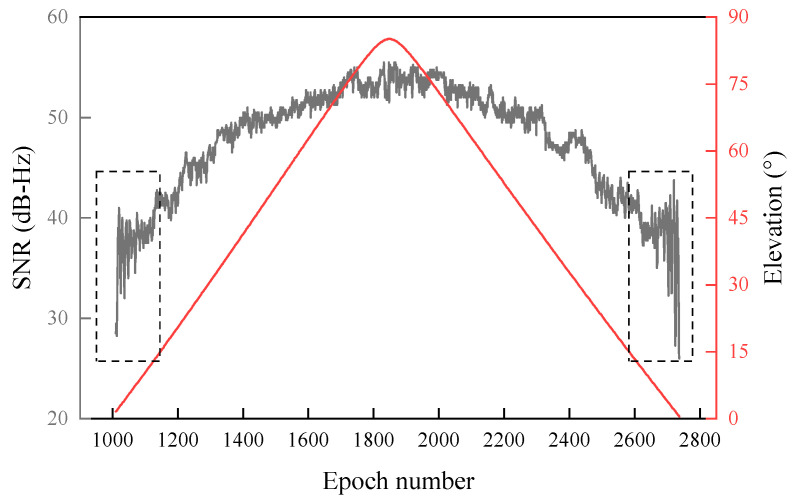
Variation of SNR and elevation angle of PRN 22 satellite on day of year (DOY) 173 in 2015. The black and red lines indicate the SNR and satellite-elevation variation with time, respectively, and the dashed boxes indicate SNR values for elevation angles of 0.5°~15°.

The interference signal composed of the direct and reflected signal is recorded by the receiver in SNR, which can be described as [20]:

(2) $${SNR}^{2}=A_{d}^{2}+A_{m}^{2}+2A_{d}A_{m}\cos\psi$$

where $A_{d}$ and $A_{m}$ are the direct and reflected signal amplitudes, respectively, and ψ is the phase difference between the direct and reflected signal. To ensure accurate positioning, the antenna of a standard geodetic receiver is typically designed with specific features to mitigate the impact of the multipath effect, thus allowing the received signal to satisfy the relationship $A_{d}\gg A_{m}$. Consequently, the SNR series shows a parabolic trend in the whole by the direct signal. In contrast, the reflected signal leads to local periodic oscillation (Figure 3), in which the information on the reflecting surface is hidden [38].

SNR is a quantitative index to evaluate signal quality [39,40]. As shown in the dashed box in Figure 3, the SNR values are smaller at low-elevation angles. They are more affected by multipath, and the periodic oscillations are more significant. Therefore, extracting the characteristic parameters of SNR oscillations for low-elevation angles is more accessible than for high-elevation angles. Although the SNR series with a larger elevation range contains more complete reflector information, the effective information is often submerged in complex noise and difficult to extract accurately. In traditional GNSS-IR models, a quadratic polynomial is used to fit the trend of the SNR arc. Then the fitting term is subtracted from the SNR arc to eliminate direct signals and a small number of reflected signals, namely the term $A_{d}^{2}+A_{m}^{2}$ in Equation (2). The oscillation term of SNR obtained can be approximated by the cosine model [10]:

(3) $${SNR}_{m}=2A_{d}A_{m}\cos\psi =A\cos\left(2\pi fx+\phi \right)$$

where A is the signal amplitude, f is the oscillation frequency, and ϕ is the phase. From the additional distance D, the phase difference ψ between the direct and reflected signal can be calculated as follows:

(4) $$\psi =\frac{2\pi }{\lambda }D=\frac{4\pi h}{\lambda }\sin\left(\theta \right)$$

where λ is the carrier wavelength. According to Equation (4), there is a linear relationship between the phase difference ψ and the sine value $\sin\left(\theta \right)$ of the satellite-elevation angle, thus:

(5) $$2\pi f=\frac{4\pi h}{\lambda }$$

After Equation (5) is simplified, the relationship between the reflector height h and frequency f of ${SNR}_{m}$ can be obtained as follows:

(6) $$h=\frac{\lambda f}{2}$$

The oscillation term varying with $\sin\left(\theta \right)$ is a non-equidistant series, so it is difficult to use the fast Fourier transform (FFT) for spectral analysis [41,42]. The conventional sea-level estimation model uses LSP to extract the frequency information of the oscillation term. The frequency f corresponding to the maximum peak amplitude in the periodogram is converted into the reflector height h according to Equation (6), and then the sea surface height is obtained by unifying reflector height to the same datum of TG.

### 3.2. Signal Decomposition Based on LMD

According to the basic principle of GNSS-IR introduced in Section 3.1, the accurate acquisition of reflection signals is a crucial step in retrieving sea-level height. For the LSP, the input signal must have a zero-mean value, and the noise level of the signal has a significant impact on frequency extraction [28,29]. Low-order polynomials cannot accurately depict the trend change in the SNR arc, and the acquired oscillation term contains a lot of noise, which makes the frequency acquired by LSP uncertain [43]. Therefore, the GNSS-IR technique typically uses SNR series with a low-elevation range to reduce the noise source. In addition, the RH range is set according to the actual sea-level variation during retrieval, and the maximum peak amplitude is retrieved only within this range to avoid abnormal values. Although these limitations improve retrieval accuracy to some extent, they also remarkably reduce the adaptability and flexibility of the technology. As an adaptive signal analysis method, LMD can decompose a complex signal into a series of signal components with different frequencies, which has a significant effect on the processing of non-stationary signals. Therefore, this paper proposes using LMD to process the SNR arc and separate the trend item and noise components from the SNR arc to provide a more stationary and purer oscillation term for LSP.

In 2005, Smith et al. proposed a new adaptive signal analysis based on EMD, namely LMD, and successfully applied it to the processing of electroencephalogram (EEG) signals [44]. LMD can decompose a complex amplitude and frequency modulation (AM-FM) signal into a series of product functions (PF), each being the product of a local envelope and a pure frequency-modulated signal. The traditional LMD algorithm uses adjacent extreme points to calculate the local mean and local envelope. It uses the moving average algorithm to process the local mean and local envelope to construct the local mean function and envelope estimation function. Then, the local mean function is separated from the original signal to obtain the zero-mean signal, and the envelope estimation function is used to demodulate the zero-mean signal. This procedure is repeated for the demodulated signal until a purely FM signal is obtained. At this time, the product of a series of envelope estimation functions obtained in the demodulation process is the final envelope signal, and the envelope signal is multiplied by the purely FM function to obtain the first PF component. After subtracting the PF component from the original signal, the entire process is repeated for the residual signal until a monotone residual signal is produced. If the original signal is designated as $x\left(t\right)$, the decomposition outcome of LMD can be represented as [44]:

(7) $$x\left(t\right)=\sum_{i=1}^{k}{PF}_{i}\left(t\right)+u_{k}\left(t\right)$$

where $u\left(t\right)$ is the monotone residual term. The traditional LMD based on the moving average algorithm is inefficient and may not be able to obtain the convergent envelope estimation function. In response, Hu et al. proposed interpolating upper and lower extremum points with cubic spline to obtain the envelope. The experimental results showed that the decomposition effect of the LMD algorithm based on cubic spline interpolation is better than that of the traditional LMD algorithm [45]. However, the envelope estimation method based on the interpolation algorithm still makes it difficult to obtain an accurate envelope. To this end, Jia et al. proposed an Empirical Optimal Envelope (EOE). The method uses tangent points instead of extreme points, approximates the optimal interpolation point position through an iterative greedy algorithm, and optimizes the envelope distance to obtain the optimal envelope. The experimental results showed that the EOE-LMD algorithm could obtain more accurate envelopes and signal components [46]. In this study, all the LMD algorithms used are in the EOE-LMD algorithm.

## 4. Sea-Level Estimation

**Figure 4:**
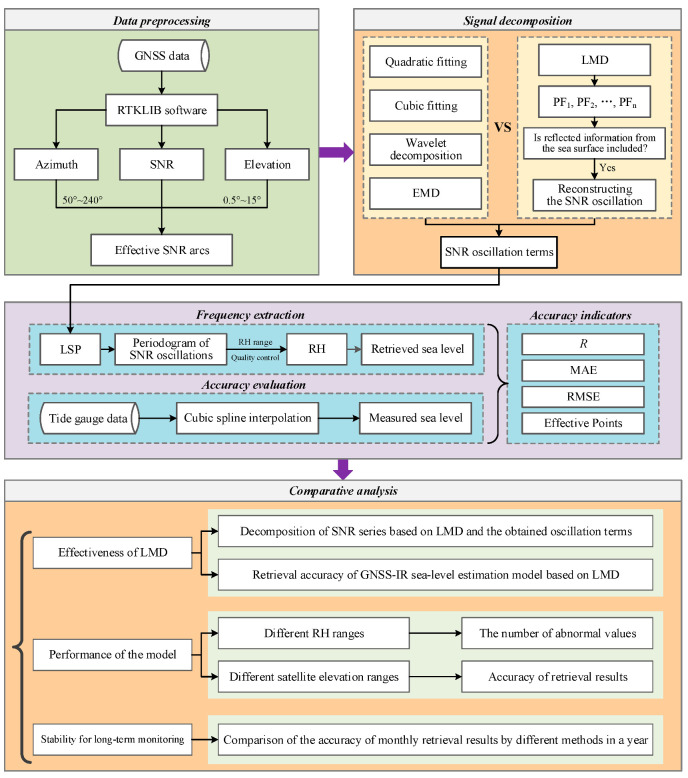
Flowchart of experiments and analysis of GNSS-IR sea-level estimation model based on LMD.

According to the theory introduced in Section 3, this study combines LMD and LSP to construct a GNSS-IR sea-level estimation model based on LMD. The experimental process is mainly divided into five steps (Figure 4).

**Step 1:** Data preprocessing. The GNSS data were processed using RTKLIB ver. 2.42 software to obtain SNR, azimuth, and satellite-elevation data. Due to the site environment, not all SNR data come from the sea surface. Generally, the effectiveness of the SNR arc can be judged according to the azimuth and the satellite elevation. SNR arcs boasting over 100 observations within the range of azimuth 50°~240° and satellite elevation 0.5°~15° were chosen for subsequent processing.

**Step 2:** Signal decomposition. The SNR arc was decomposed by LMD, and the signal components containing sea surface information were used to construct the oscillation term. Concurrently, this study utilized quadratic fitting, cubic fitting, wavelet decomposition, and EMD for SNR arc processing. Their retrieval results were compared to analyze the effectiveness and accuracy of LMD.

**Step 3:** Frequency extraction. LSP was used to extract the effective frequency in the SNR oscillation term. The frequency was converted to RH according to Equation (6), and then the RH was converted to the sea surface height under the same datum of TG. This study retrieved RH in the range of 4~7 m, and a wider RH range was also used in exploring the performance of LMD. In addition, the retrieval values of peak amplitude less than 3 and peak-to-noise ratio (the ratio of the maximum peak amplitude to the average peak amplitude of noise in the RH range) less than 2 were removed [40].

**Step 4:** Accuracy evaluation. The measured sea-level height at the corresponding time was obtained by interpolating the TG data using the cubic spline method. The gross errors in retrieval results were removed based on the principle of double or triple standard deviation. The correlation coefficient (Pearson correlation coefficient, R), RMSE, and mean absolute error (MAE) between the retrieved and measured values were calculated to evaluate the accuracy of retrieval results.

**Step 5:** Comparative analysis. This study first used SNR data for 7 consecutive days to verify the effectiveness of LMD. On this basis, the performance of the GNSS-IR sea-level estimation model based on LMD and the influence of noise on retrieval results were explored from two aspects of different RH ranges and satellite-elevation ranges, respectively. Finally, the data for one consecutive year were processed to verify the stability of the model in long-term sea-level monitoring.

## 5. Results and Discussion

### 5.1. Decomposition Results of LMD for SNR

**Figure 5:**
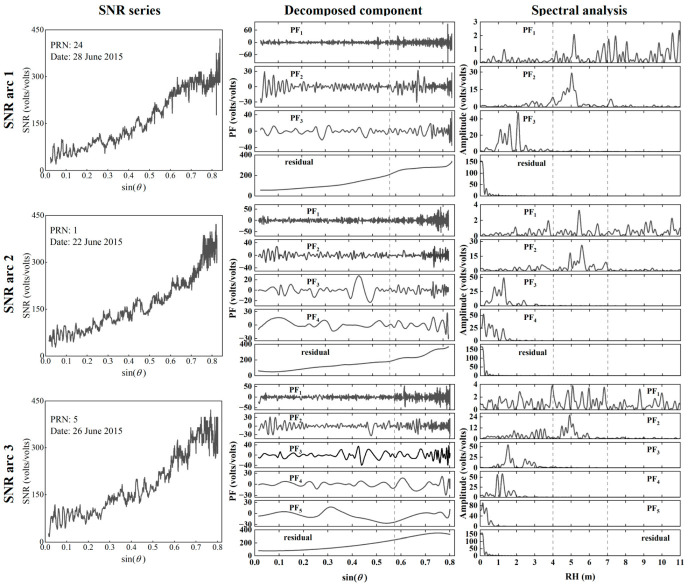
Decomposition results of three SNR arcs. Rows: (**top**) SNR arc 1; (**middle**) SNR arc 2; (**bottom**) SNR arc 3. Columns: (**left**) SNR series variation; (**middle**) Components obtained by decomposition; (**right**) Spectral analysis of each component. Reference lines (dashed black lines) are added to the sine at 35° elevation and RH positions at 4 m and 7 m.

The key to using LMD to extract SNR oscillation is to find the component where reflected information from the sea surface is located. For this purpose, SNR data from DOY 173–179 in 2015 at the SC02 site were processed using LMD. From the examined data over seven consecutive days, there were 380 eligible SNR arcs, the majority of which were decomposed into three to five layers of PF components (as shown in Table 1). Figure 5 shows the decomposition results of three different SNR arcs, which were processed by LMD to obtain the three, four, and five layers of PF components, respectively, and the periodogram of each component is presented. As shown in the figure, although the decomposition layers of different SNR arcs are different, the significant periodic fluctuations are concentrated in PF_1_ and PF_2_. Furthermore, their periodograms exhibit significant peaks within the RH range of 4~7 m. The RH corresponding to the maximum peak amplitude of PF_2_ falls within this range. In contrast, other components show scant and discontinuous fluctuations in the low-elevation range, and the RH corresponding to the significant peak in the periodogram is less than 4 m. In addition, the spectral analysis results of the residual term are also given in the figure. It can be seen that the residual term has essentially the same trend as the SNR arc, which shows a significant peak near the zero axis in the periodogram.

**Figure 6:**
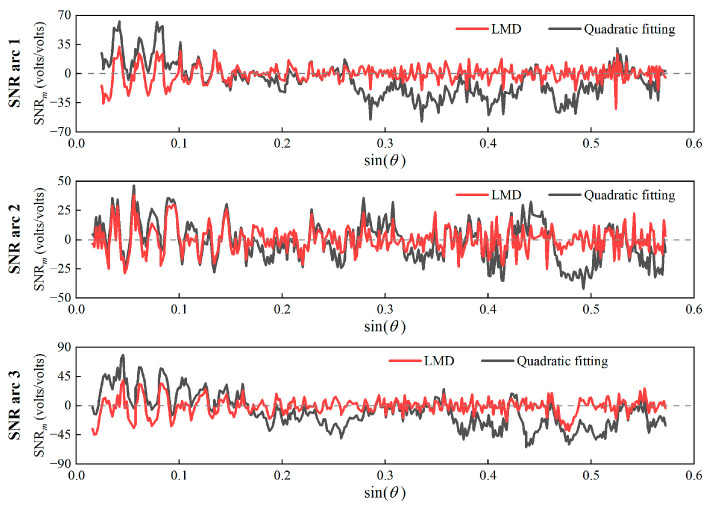
The oscillation terms obtained by LMD and quadratic fitting for different arcs. (**Top**) SNR arc 1; (**Middle**) SNR arc 2; (**Bottom**) SNR arc 3.

The sea-level variations were estimated using the PF_1_, PF_2_, PF_3_, and PF_4_ of different SNR arcs, respectively, and the results are shown in Table 2. It can be seen that the retrieval results of PF_1_ and PF_2_ have better correlation and accuracy with TG data than other PF components. Accordingly, PF_1_ and PF_2_ were selected to construct the oscillation term, which was then compared to quadratic fitting. The oscillation terms obtained by LMD are more regular and stationary (Figure 6).

**Figure 7:**
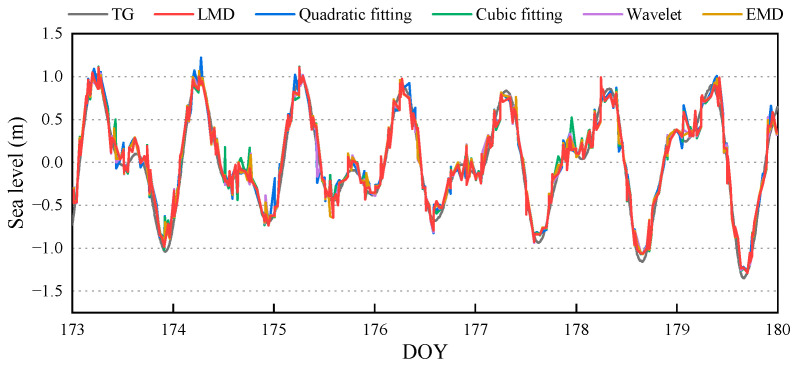
Sea-level retrieval results for 7 consecutive days by different methods. The SNR arc was decomposed by 6 layers using Daubechies4 wavelet, and 1–4 layers of components were selected to construct the oscillation term. EMD is an adaptive signal-decomposition method. When it is used to process SNR arc, 5–6 layers of intrinsic mode functions (IMF) are generally obtained, and 1–3 layers of IMF components were selected to construct oscillation term.

Following the experimental results, the oscillation term comprising ${PF}_{1}+{PF}_{2}$ was selected to retrieve the sea-level height, and the retrieval results are presented in Figure 7 and Table 3. Additionally, the component combination schemes of wavelet decomposition and EMD were obtained through many experiments. On the premise of ensuring a sufficient number of retrieval values, the combination of the components with the best accuracy in the experimental results was selected as the effective component.

As seen from Figure 7, the R between the retrieval results of different methods and measured values all reaches 0.97. Moreover, the number of retrieval values exceeded 350, with daily averages surpassing 50. The overall trend of retrieval results can better reflect the actual sea-level variation. As shown in Table 3, in descending order of accuracy: LMD > wavelet decomposition > EMD > cubic fitting > quadratic fitting. The accuracy of LMD, wavelet decomposition, and EMD is significantly better than that of low-order polynomials. Cubic fitting shows a slight improvement in accuracy compared to quadratic fitting, and the accuracy of the LMD is slightly better than that of the wavelet decomposition and EMD, although the difference is negligible. The RMSE and MAE of the new model based on LMD were 11.74 cm and 9.30 cm, improving 11.99% and 12.51% over the quadratic fitting, respectively.

### 5.2. Performance Analysis of Different RH Ranges

**Figure 8:**
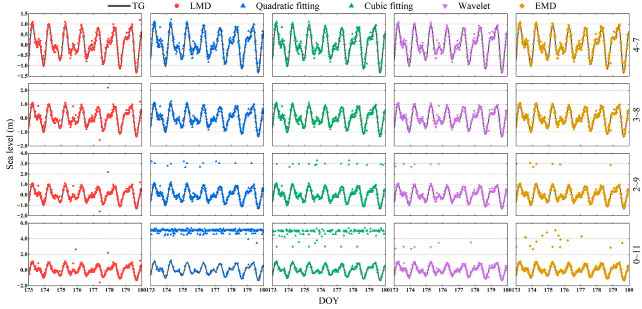
Retrieval results of the five methods in different RH ranges. Rows: from top to bottom are the RH ranges of 4~7 m, 3~8 m, 2~9 m, and 0~11 m, respectively. Columns: from left to right are the retrieval results of LMD, quadratic fitting, cubic fitting, wavelet decomposition, and EMD, respectively.

To compare the retrieval results of different RH ranges, four RH ranges of 4~7 m, 3~8 m, 2~9 m, and 0~11 m were set in the retrieval. Figure 8 shows the retrieval results of different methods that meet the quality control conditions before removing abnormal values.

As seen in Figure 8, the retrieval results of quadratic fitting stay mostly the same when the RH range is expanded from 4~7 m to 3~8 m. When the RH range is expanded to 2~9 m, a small number of abnormal values appear in the retrieval results. These values are approximately 3 m, which is above sea level and approximate to the ground height. The corresponding frequency represents the reflected information from the ground, and it shows in the periodogram that the peak of the reflected signal from the ground replaces the one from the sea surface as the main peak. As the RH range broadens to 0~11 m, more abnormal values appear in the retrieval results. Their height is concentrated around 5 m, close to the antenna height. The corresponding frequency is close to the zero axis in the periodogram, which represents the residual trend term in the oscillation term, and the peak near the zero axis replaces that of the reflected signal from the sea surface as the main peak. For LMD, when the RH range is expanded from 4~7 m to 3~8 m, the abnormal values appearing in the retrieval results are sparse and distributed. This outcome arises because the effective frequencies of some SNR series reside in higher-order components, which are eliminated as noise, thus causing the loss of effective information and an error in frequency retrieval. As the RH range continues to be expanded, the retrieval results of LMD change little, and it can maintain a high retrieval accuracy and stability all the time. With the expansion of the RH range, the change in retrieval results obtained by cubic fitting is consistent with that of the quadratic fitting. However, when the RH range is extended to 0~11 m, the number of abnormal values in the cubic fitting is significantly reduced compared to the quadratic fitting. The wavelet decomposition and EMD greatly reduce the number of abnormal values compared with the low-order polynomials, but a small number of abnormal values still appear when the RH range is extended to 0~11 m. This indicates that the wavelet decomposition and EMD can also effectively remove the influence of noise in the SNR series. However, some SNR oscillation terms still contain ground information, resulting in certain abnormal values in retrieval results. To illustrate the differences in accuracy and the number of points between different methods, Table 4 lists the accuracy evaluation of retrieval results after removing abnormal values.

Table 4 shows that the new model based on LMD always obtains retrieval results with high accuracy and stable quantity in different RH ranges. LMD can effectively remove the interference of low-frequency noise. Compared with wavelet decomposition and EMD, LMD boasts superior signal decomposition, accurately distinguishing noise from the oscillation term and efficiently circumventing abnormal values.

**Figure 9:**
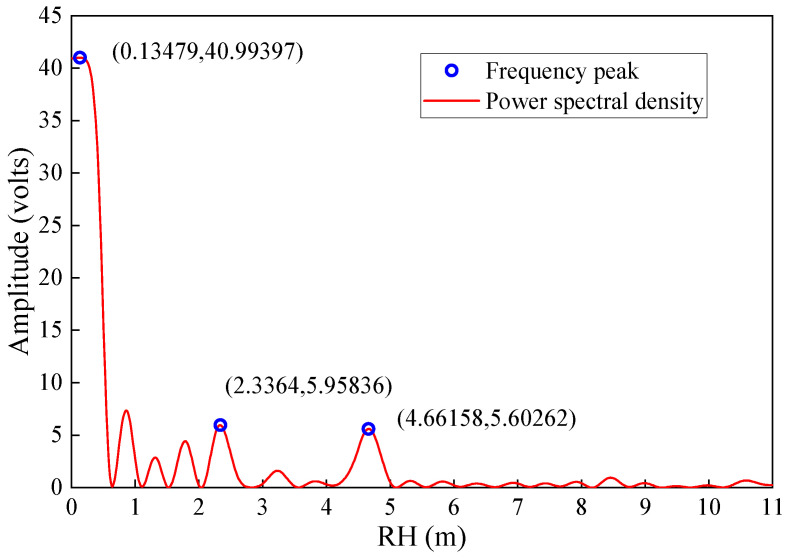
Periodogram of the oscillation term. The frequency of the horizontal axis in the periodogram is converted to RH. The blue circle represents the maximum peak amplitude retrieved in the RH ranges of 4~7 m, 2~9 m, and 0~11 m, respectively, and the coordinate position of the peak is marked.

Due to the poor fitting effect of the quadratic polynomial, the obtained oscillation term often contains a residual trend term. Furthermore, when the SNR series contains noise from other reflecting surfaces, it cannot be removed by quadratic fitting. An oscillatory term containing a trend term and noise exhibits multiple significant peaks in the periodogram (Figure 9). Setting a range for retrieving RH can reduce the interference of other frequency components in the SNR oscillation to a certain extent and avoid abnormal values. However, it is essentially just a constrained retrieval condition. The existence of noise will still impact the accuracy and stability of identified frequency, resulting in low-accuracy retrieval results, as confirmed by the experimental outcomes presented in Section 5.2. In addition, it can be seen from the experimental results that the height of most anomalous results is greater than that of sea level, and only a few anomalous values are lower than sea level due to the loss of effective information. This fact shows that the high-frequency noise in the SNR series has no direct effect on frequency extraction.

### 5.3. Performance Analysis of Different Elevation Ranges

**Figure 10:**
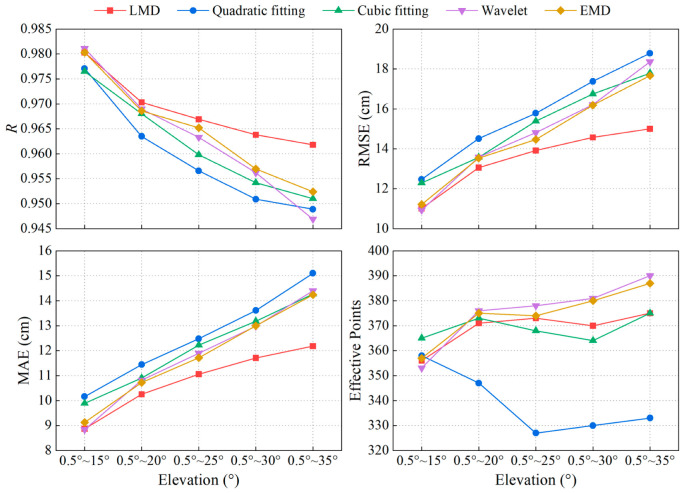
Accuracy comparison of retrieval results by five methods in different elevation ranges. (**Top left**) R between the retrieval results of five methods in different elevation ranges and TG data; (**Top right**) RMSE; (**Bottom left**) MAE; (**Bottom right**) The number of retrieval values by five methods in different elevation ranges.

To explore the retrieval performance of the new model based on LMD at high elevation, SNR series of 0.5°~15°, 0.5°~20°, 0.5°~25°, 0.5°~30°, and 0.5°~35° were used to retrieve sea-level height, respectively. The retrieval results are illustrated in Table 5 and Figure 10.

Combining Table 5 and Figure 10, it can be found that the accuracy of the retrieval results obtained by different methods drops as the elevation range widens. Except for quadratic fitting, the number of retrieved values remains stable for other methods. However, in this process, the accuracy of LMD is always better than the other four methods.

**Figure 11:**
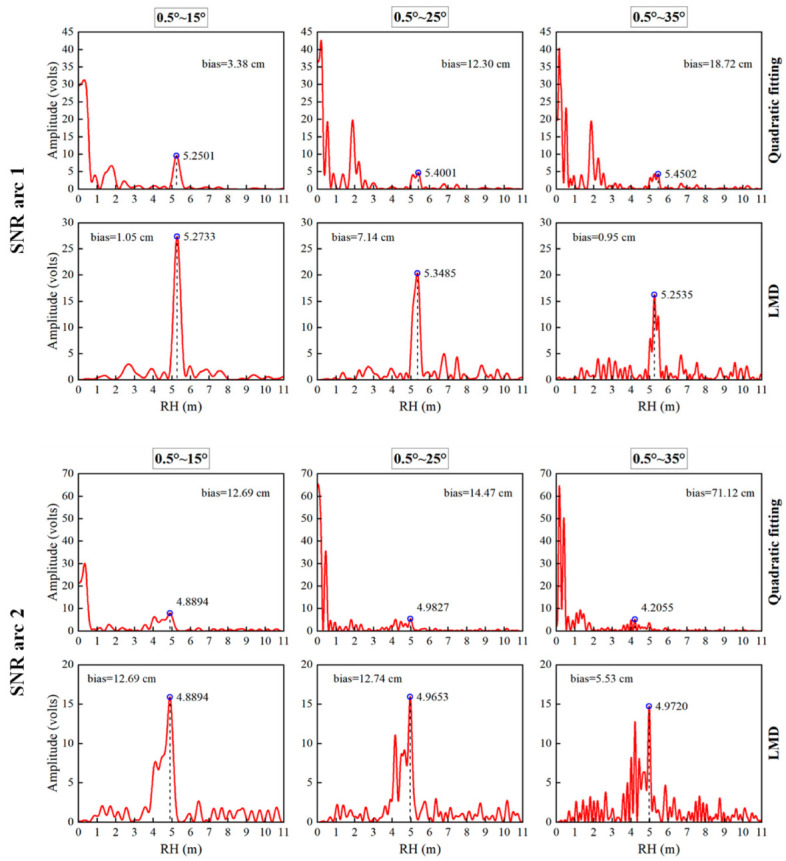
Periodograms of the oscillation terms at different elevation angles (**Columns**) obtained by different methods (**Rows**) for two SNR arcs. (**Top**) SNR arc 1, (**Bottom**) SNR arc 2. Each picture shows the variation of power spectral density with RH (red line) and the maximum peak amplitude retrieved in the range of 4~7 m (blue circles). The absolute deviation between the retrieval and measured value at the corresponding time is marked in the figure (unit: cm).

For LSP, the uncertainty of the frequency is inversely proportional to the number of samples [47]. As the elevation range widens, the length of the SNR series input to LSP increases, which is advantageous for the solution of the frequencies. Sea-level estimation based on SNR analysis attempts to find the average frequency of the whole series. It uses the sea-level height transformed from this frequency to represent the sea-level height at the middle moment of the entire series. LSP can only identify significant fluctuations in the series, and the height obtained may be the sea-level height at any time in the observation period. When the SNR series with a wider elevation range retrieves sea-level height, the sea-level variation is more significant over a longer observation time, decreasing retrieval accuracy. Figure 11 displays the spectral analysis results for two SNR arcs with varying elevation ranges, processed using quadratic fitting and LMD, respectively. The first one produced an abnormal value in the elevation range of 0.5°~35° by quadratic fitting, while LMD obtained an effective reversal result. The second one obtained effective retrieval results at the high-elevation range after processing by both methods, but the retrieval value of LMD was more accurate. It can be found from Figure 11 that with the expansion of the elevation range, the fitting deviation of the quadratic polynomial increases, and the trend term contained in the oscillation term greatly reduces the significance of the main peak. When the elevation angle is greater than 15°, the interference components contained in the SNR series become more complex, and the number of peaks with similar frequencies increases in the periodogram. For LSP, the significant trend items and complex noise in the signal intensify the uncertainty of the obtained frequency, which may increase the deviation between the obtained and actual frequency and reduce the accuracy of the retrieval results. The noise peak may even replace the peak of RH as the main peak, resulting in abnormal values. In addition, LMD can only remove part of the noise signals far from RH and cannot change the frequency components in the RH range, and the influence of noise components in the range on retrieving RH still exists.

### 5.4. Stability of Long-Term Monitoring

**Figure 12:**
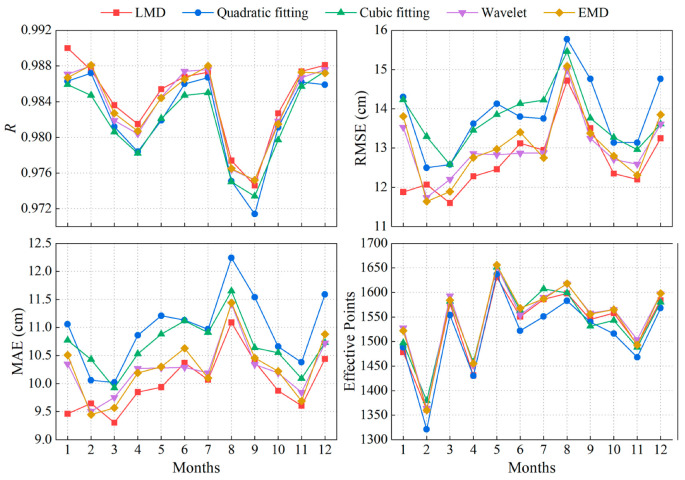
Accuracy comparison of monthly retrieval results by different methods. (**Top left**) R between the monthly obtained retrieval results by different methods and TG data; (**Top right**) RMSE; and (**Bottom left**) MAE; (**Bottom right**) The number of monthly obtained retrieval values by different methods.

As seen from the previous experiments, LMD can effectively weaken the influence of noise and still have excellent performance in a wider RH range. The sea-level variation at Friday Harbor is greater than 3 m during a year, and the RH range should be appropriately expanded during retrieval to meet the complex sea-level variation. To further verify the stability of the new model based on LMD for long-term monitoring, one year of SNR data from the SC02 site in 2015 was processed using the model. The accuracy of the monthly retrieval results was assessed individually and is presented in both Table 6 and Figure 12.

As seen from Table 6 and Figure 12, among the retrieval results of 12 consecutive months, there are 8 months in which the accuracy index of LMD is entirely superior to the other four methods. The average accuracy of monthly retrieval results is also better than the other four methods. The number of retrieval values from different methods remained basically at the same level. Moreover, the retrieval result accuracy of LMD, wavelet, and EMD surpasses that of low-order polynomials, barring the month of December. Compared with the retrieval results of quadratic fitting, the RMSE of LMD is minimally improved in February at 3.44% and maximally improved in January at 16.92%, and the MAE is minimally improved in February at 4.12% and maximally improved in January at 14.45%. The RMSE and MAE of monthly retrieval results are improved by 8.34% and 8.87% on average. Over the one-year retrieval results, the GNSS-IR sea-level estimation model based on LMD shows high accuracy and excellent performance.

## 6. Conclusions

This study developed a GNSS-IR sea-level estimation model based on LMD. In this model, the SNR arc was decomposed by LMD, and the signal components containing reflected information from the sea surface were selected to construct the oscillation term. LMD provides a lower noise oscillation term for LSP compared to quadratic fitting, therefore effectively enhancing retrieval accuracy. The results show that the new model based on LMD still has excellent performance under loose constraints, which is of great significance for sea-level monitoring in the long term and without a priori tide-level height. In the one-year retrieval results, the GNSS-IR model for sea-level estimation based on LMD shows excellent retrieval accuracy and stability.

Changes in RH range and satellite-elevation range affect retrieval results differently. As the RH range is expanded, more noise peaks are included in the retrieval range in the periodogram. An anomalous retrieval result is obtained when the peak amplitude of the noise component is larger than that of the RH. In contrast, the use of SNR arcs with a high satellite-elevation range reduces the significance of the effective peaks and produces more noise peaks with similar frequencies, reducing the accuracy of the retrieval results. The improved method based on signal decomposition can effectively remove the trend term and the noise outside the RH range in the SNR series, which is the reason for its ability to maintain high retrieval accuracy and a sufficiently large number of retrieved values despite the loose constraints (either in the wide RH range or satellite-elevation angle range). LMD shows significant advantages over other signal separation methods with its excellent signal-decomposition performance. Unfortunately, LMD cannot change the frequency components in the RH range, which is a limitation of the LMD method and all signal analysis methods, which is consistent with the experimental findings of Wang et al. [31].

The retrieval process requires other parameter settings to ensure retrieval result accuracy, such as data length, azimuth range, peak-to-noise ratio, and peak amplitude power. In the comparison experiments about different elevation ranges, the numerical advantage of points in our results is not as significant as that of Zhang et al. [32]. This is mainly due to the different criteria for determining the effective arc. Therefore, the effect of these parameters on retrieval accuracy is equally worth investigating. This paper used fixed two-layer PF components to construct the oscillatory term. LMD is an adaptive signal-decomposition method, and the number of decomposition layers varies for different SNR series. The combination of fixed components can easily cause the loss of effective information, which is unreasonable. However, the existing dynamic selection method depends on the oscillation term obtained by quadratic fitting and cannot be carried out independently. Moreover, it cannot accurately judge the boundary between the trend item and the effective component, which fails to exploit the advantages of the signal-decomposition method fully. In the future, the problem will continue to be studied by combining the adaptive layer selection method with LMD to improve the stability of the method further. In addition, the data union of multi-systems can greatly improve the temporal resolution of retrieval results, but the SNR data of different systems with different frequencies have different sensitivities to noise, and the direct combination cannot significantly improve the accuracy level of retrieval results. Therefore, we hope to improve the retrieval accuracy of multi-mode and multi-frequency by way of combining them after denoising.

## Figures and Tables

**Table 1:** Statistical results of decomposition layers for all effective arcs.

Number of PF Components	Number of SNR Series
3	35
4	256
5	81
6	7
7	1

**Table 2:** The retrieval results of different PF components.

Results	PF_1_	PF_2_	PF_3_	PF_4_
R	0.7252	0.9525	0.0866	1.0000
RMSE (cm)	47.39	17.27	109.92	108.47
MAE (cm)	28.16	11.90	84.96	108.41
Effective Points	115	348	62	2

**Table 3:** Accuracy comparison of results obtained by different methods. The RH range was set to 4~7 m, and the gross errors larger than the triple standard deviation in the search results were removed.

Methods	R	RMSE (cm)	MAE (cm)	Effective Points
LMD	0.9781	11.74	9.30	363
Quadratic fitting	0.9734	13.34	10.63	364
Cubic fitting	0.9740	12.99	10.28	370
Wavelet	0.9775	11.94	9.47	364
EMD	0.9778	11.96	9.59	365

**Table 4:** Accuracy evaluation of retrieval results in different RH ranges for five methods. Abnormal values retrieved outside the RH range of 4~7 m and gross errors larger than the double standard deviation in the retrieval results were removed.

Results	Methods	4~7 m	3~8 m	2~9 m	0~11 m
R	LMD	0.9803	0.9774	0.9774	0.9774
	Quadratic fitting	0.9771	0.9770	0.9764	0.9844
	Cubic fitting	0.9765	0.9769	0.9760	0.9791
	Wavelet	0.9811	0.9783	0.9796	0.9770
	EMD	0.9803	0.9803	0.9803	0.9805
RMSE (cm)	LMD	11.01	12.00	11.99	11.99
	Quadratic fitting	12.47	12.49	12.51	12.39
	Cubic fitting	12.29	12.22	12.28	11.22
	Wavelet	10.93	11.69	11.29	12.09
	EMD	11.22	11.22	11.09	11.04
MAE (cm)	LMD	8.87	9.44	9.44	9.44
	Quadratic fitting	10.16	10.18	10.25	10.31
	Cubic fitting	9.89	9.87	9.93	9.26
	Wavelet	8.84	9.33	9.11	9.61
	EMD	9.13	9.13	9.06	9.01
Effective Points	LMD	356	366	367	367
	Quadratic fitting	358	359	349	114
	Cubic fitting	365	367	353	222
	Wavelet	353	363	354	361
	EMD	357	359	350	343

**Table 5:** Accuracy evaluation of retrieval results of five methods in different elevation ranges. The gross errors in retrieval results were eliminated based on the double standard deviation.

Results	Methods	0.5°~15°	0.5°~20°	0.5°~25°	0.5°~30°	0.5°~35°
R	LMD	0.9803	0.9703	0.9669	0.9638	0.9618
	Quadratic fitting	0.9771	0.9635	0.9566	0.9509	0.9489
	Cubic fitting	0.9765	0.9680	0.9598	0.9542	0.9510
	Wavelet	0.9811	0.9690	0.9633	0.9562	0.9469
	EMD	0.9803	0.9686	0.9652	0.957	0.9524
RMSE (cm)	LMD	11.01	13.06	13.91	14.57	15.00
	Quadratic fitting	12.47	14.51	15.78	17.38	18.79
	Cubic fitting	12.29	13.57	15.38	16.75	17.79
	Wavelet	10.93	13.58	14.81	16.21	18.36
	EMD	11.22	13.53	14.46	16.18	17.67
MAE (cm)	LMD	8.87	10.25	11.06	11.71	12.18
	Quadratic fitting	10.16	11.44	12.48	13.61	15.10
	Cubic fitting	9.89	10.90	12.22	13.18	14.27
	Wavelet	8.84	10.82	11.90	12.98	14.40
	EMD	9.13	10.72	11.72	13.00	14.24
Effective Points	LMD	356	371	373	370	375
	Quadratic fitting	358	347	327	330	333
	Cubic fitting	365	373	368	364	375
	Wavelet	353	376	378	381	390
	EMD	357	375	374	380	387

**Table 6:** Accuracy statistics of monthly retrieval results by different methods. The RH range was set to 3~8 m, the gross errors larger than the double standard deviation were removed from the retrieval results, and the accuracy of retrieval results was evaluated each month.

Months	Methods	R	RMSE (cm)	MAE (cm)	Effective Points
Jan	LMD	0.9900	11.88	9.46	1478
	Quadratic fitting	0.9863	14.30	11.06	1488
	Cubic fitting	0.9859	14.23	10.77	1497
	Wavelet	0.9871	13.53	10.35	1528
	EMD	0.9867	13.81	10.51	1522
Feb	LMD	0.9875	12.07	9.65	1361
	Quadratic fitting	0.9872	12.50	10.06	1321
	Cubic fitting	0.9847	13.29	10.43	1379
	Wavelet	0.9880	11.74	9.51	1364
	EMD	0.9881	11.64	9.45	1360
Mar	LMD	0.9836	11.60	9.30	1577
	Quadratic fitting	0.9812	12.58	10.02	1554
	Cubic fitting	0.9806	12.58	9.92	1582
	Wavelet	0.9819	12.20	9.75	1593
	EMD	0.9827	11.89	9.57	1584
Apr	LMD	0.9815	12.28	9.85	1431
	Quadratic fitting	0.9784	13.62	10.86	1430
	Cubic fitting	0.9782	13.45	10.53	1458
	Wavelet	0.9804	12.86	10.27	1451
	EMD	0.9807	12.76	10.19	1455
May	LMD	0.9854	12.46	9.94	1631
	Quadratic fitting	0.9819	14.13	11.21	1637
	Cubic fitting	0.9821	13.85	10.88	1651
	Wavelet	0.9845	12.83	10.28	1653
	EMD	0.9844	12.97	10.30	1656
Jun	LMD	0.9868	13.12	10.37	1551
	Quadratic fitting	0.9860	13.80	11.13	1522
	Cubic fitting	0.9847	14.13	11.12	1563
	Wavelet	0.9874	12.87	10.29	1554
	EMD	0.9865	13.40	10.63	1568
Jul	LMD	0.9873	12.95	10.07	1586
	Quadratic fitting	0.9867	13.75	10.97	1551
	Cubic fitting	0.9850	14.22	10.91	1607
	Wavelet	0.9876	12.88	10.19	1590
	EMD	0.9880	12.75	10.10	1587
Aug	LMD	0.9774	14.72	11.09	1598
	Quadratic fitting	0.9751	15.77	12.24	1583
	Cubic fitting	0.9750	15.46	11.65	1599
	Wavelet	0.9764	14.99	11.42	1617
	EMD	0.9765	15.09	11.44	1618
Sep	LMD	0.9746	13.51	10.39	1545
	Quadratic fitting	0.9714	14.76	11.54	1540
	Cubic fitting	0.9734	13.76	10.64	1531
	Wavelet	0.9752	13.25	10.34	1558
	EMD	0.9752	13.38	10.46	1556
Oct	LMD	0.9827	12.35	9.87	1558
	Quadratic fitting	0.9811	13.14	10.66	1516
	Cubic fitting	0.9797	13.27	10.55	1543
	Wavelet	0.9818	12.71	10.19	1565
	EMD	0.9815	12.80	10.22	1565
Nov	LMD	0.9874	12.20	9.61	1494
	Quadratic fitting	0.9862	13.14	10.38	1468
	Cubic fitting	0.9857	12.96	10.09	1488
	Wavelet	0.9867	12.59	9.84	1504
	EMD	0.9873	12.31	9.69	1493
Dec	LMD	0.9881	13.25	10.44	1584
	Quadratic fitting	0.9859	14.76	11.59	1568
	Cubic fitting	0.9874	13.61	10.73	1580
	Wavelet	0.9876	13.61	10.72	1597
	EMD	0.9872	13.85	10.88	1598

## Data Availability

Not applicable.

## References

[1] Abbass K., Qasim M.Z., Song H., Murshed M., Mahmood H., Younis I. (2022). A review of the global climate change impacts, adaptation, and sustainable mitigation measures. Environ. Sci. Pollut. Res..

[2] Melet A., Meyssignac B., Almar R., Le Cozannet G. (2018). Under-estimated wave contribution to coastal sea-level rise. Nat. Clim. Chang..

[3] Jia Y., Xiao K., Lin M., Zhang X. (2022). Analysis of Global Sea Level Change Based on Multi-Source Data. Remote Sens..

[4] Wang X., Niu Z., Chen S., He X. (2021). A Correction Method of Height Variation Error Based on One SNR Arc Applied in GNSS–IR Sea-Level Retrieval. Remote Sens..

[5] Martin-Neira M. (1993). A passive reflectometry and interferometry system (PARIS): Application to ocean altimetry. ESA J..

[6] Auber J.C., Bibaut A., Rigal J.M. Characterization of Multipath on Land and Sea at GPS Frequencies. Proceedings of the 7th International Technical Meeting of the Satellite Division of the Institute of Navigation (ION GPS 1994).

[7] Soulat F., Caparrini M., Germain O., Lopez-Dekker P., Taani M., Ruffini G. (2004). Sea state monitoring using coastal GNSS-R. Geophys. Res. Lett..

[8] Rodriguez-Alvarez N., Camps A., Vall-Llossera M., Bosch-Lluis X., Monerris A., Ramos-Perez I., Valencia E., Marchan-Hernandez J.F., Martinez-Fernandez J., Baroncini-Turricchia G. (2010). Land geophysical parameters retrieval using the interference pattern GNSS-R technique. IEEE Trans. Geosci. Remote Sens..

[9] Rius A., Nogués-Correig O., Ribó S., Cardellach E., Oliveras S., Valencia E., Park H., Tarongí J.M., Camps A., van der Marel H. (2012). Altimetry with GNSS-R interferometry: First proof of concept experiment. GPS Solut..

[10] Larson K.M., Small E.E., Gutmann E.D., Bilich A.L., Braun J.J., Zavorotny V.U. (2008). Use of GPS receivers as a soil moisture network for water cycle studies. Geophys. Res. Lett..

[11] Larson K.M., Braun J.J., Small E.E., Zavorotny V.U., Gutmann E.D., Bilich A.L. (2009). GPS multipath and its relation to near-surface soil moisture content. IEEE J. Sel. Top. Appl. Earth Obs. Remote Sens..

[12] Larson K.M., Gutmann E.D., Zavorotny V.U., Braun J.J., Williams M.W., Nievinski F.G. (2009). Can we measure snow depth with GPS receivers?. Geophys. Res. Lett..

[13] Larson K.M., Nievinski F.G. (2013). GPS snow sensing: Results from the EarthScope Plate Boundary Observatory. GPS Solut..

[14] Lv J., Zhang R., Tu J., Liao M., Pang J., Yu B., Li K., Xiang W., Fu Y., Liu G. (2021). A GNSS-IR Method for Retrieving Soil Moisture Content from Integrated Multi-Satellite Data That Accounts for the Impact of Vegetation Moisture Content. Remote Sens..

[15] Liang Y., Lai J., Ren C., Lu X., Zhang Y., Ding Q., Hu X. (2022). GNSS-IR Multisatellite Combination for Soil Moisture Retrieval Based on Wavelet Analysis Considering Detection and Repair of Abnormal Phases. Measurement.

[16] Peng D., Hill E.M., Li L., Switzer A.D., Larson K.M. (2019). Application of GNSS interferometric reflectometry for detecting storm surges. GPS Solut..

[17] Larson K.M., Lay T., Yamazaki Y., Cheung K.F., Ye L., Williams S.D., Davis J.L. (2021). Dynamic sea level variation from GNSS: 2020 Shumagin earthquake tsunami resonance and Hurricane Laura. Geophys. Res. Lett..

[18] Zhang S., Wang T., Wang L., Zhang J., Peng J., Liu Q. (2021). Evaluation of GNSS-IR for retrieving soil moisture and vegetation growth characteristics in wheat farmland. J. Surv. Eng..

[19] Sui M., Chen K., Shen F. (2022). Monitoring of Wheat Height Based on Multi-GNSS Reflected Signals. Remote Sens..

[20] Larson K.M., Löfgren J.S., Haas R. (2013). Coastal sea level measurements using a single geodetic GPS receiver. Adv. Space Res..

[21] Löfgren J.S., Haas R. (2014). Sea level measurements using multi-frequency GPS and GLONASS observations. EURASIP J. Adv. Signal Process..

[22] Larson K.M., Ray R.D., Nievinski F.G., Freymueller J.T. (2013). The accidental tide gauge: A GPS reflection case study from Kachemak Bay, Alaska. IEEE Geosci. Remote Sens. Lett..

[23] Williams S.D.P., Nievinski F.G. (2017). Tropospheric delays in ground-based GNSS multipath reflectometry—Experimental evidence from coastal sites. J. Geophys. Res. Solid Earth.

[24] Jin S., Qian X., Wu X. (2017). Sea level change from BeiDou Navigation Satellite System-Reflectometry (BDS-R): First results and evaluation. Glob. Planet. Chang..

[25] Wang X., Zhang Q., Zhang S. (2018). Azimuth selection for sea level measurements using geodetic GPS receivers. Adv. Space Res..

[26] Wang X., Zhang Q., Zhang S. (2019). Sea level estimation from SNR data of geodetic receivers using wavelet analysis. GPS Solut..

[27] Wang X., He X., Zhang Q. (2019). Evaluation and combination of quad-constellation multi-GNSS multipath reflectometry applied to sea level retrieval. Remote Sens. Environ..

[28] Purnell D., Gomez N., Chan N.H., Strandberg J., Holland D.M., Hobiger T. (2020). Quantifying the uncertainty in ground-based GNSS-reflectometry sea level measurements. IEEE J. Sel. Top. Appl. Earth Obs. Remote Sens..

[29] VanderPlas J.T. (2018). Understanding the lomb–scargle periodogram. Astrophys. J. Suppl. Ser..

[30] Bilich A., Larson K.M. (2007). Correction published 29 March 2008: Mapping the GPS multipath environment using the signal-to-noise ratio (SNR). Radio Sci..

[31] Wang X., Zhang Q., Zhang S. (2018). Water levels measured with SNR using wavelet decomposition and Lomb–Scargle periodogram. GPS Solut..

[32] Zhang S., Liu K., Liu Q., Zhang C., Zhang Q., Nan Y. (2019). Tide variation monitoring based improved GNSS-MR by empirical mode decomposition. Adv. Space Res..

[33] Hu Y., Yuan X., Liu W., Wickert J., Jiang Z., Haas R. (2021). GNSS-IR Model of Sea Level Height Estimation Combining Variational Mode Decomposition. IEEE J. Sel. Top. Appl. Earth Obs. Remote Sens..

[34] Strandberg J., Hobiger T., Haas R. (2016). Improving GNSS-R sea level determination through inverse modeling of SNR data. Radio Sci..

[35] Vu P.L., Ha M.C., Frappart F., Darrozes J., Ramillien G., Dufrechou G., Gegout P., Morichon D., Bonneton P. (2019). Identifying 2010 Xynthia storm signature in GNSS-R-based tide records. Remote Sens..

[36] Larson K.M., Ray R.D., Williams S.D.P. (2017). A 10-year comparison of water levels measured with a geodetic GPS receiver versus a conventional tide gauge. J. Atmos. Ocean. Technol..

[37] Roesler C., Larson K.M. (2018). Software tools for GNSS interferometric reflectometry (GNSS-IR). GPS Solut..

[38] Song M., He X., Wang X., Zhou Y., Xu X. (2019). Study on the quality control for periodogram in the determination of water level using the GNSS-IR technique. Sensors.

[39] Larson K.M., Small E.E., Gutmann E., Bilich A., Axelrad P., Braun J. (2008). Using GPS multipath to measure soil moisture fluctuations: Initial results. GPS Solut..

[40] Löfgren J.S., Haas R., Scherneck H.G. (2014). Sea level time series and ocean tide analysis from multipath signals at five GPS sites in different parts of the world. J. Geodyn..

[41] Lomb Nicholas R. (1976). Least-squares frequency analysis of unequally spaced data. Astrophys. Space Sci..

[42] Scargle J.D. (1982). Studies in astronomical time series analysis. II-Statistical aspects of spectral analysis of unevenly spaced data. Astrophys. J..

[43] Ran Q., Zhang B., Yao Y., Yan X., Li J. (2022). Editing arcs to improve the capacity of GNSS-IR for soil moisture retrieval in undulating terrains. GPS Solut..

[44] Smith J.S. (2005). The local mean decomposition and its application to EEG perception data. J. R. Soc. Interface.

[45] Hu J.S., Yang S.X., Ren D.Q. (2009). Spline-based local mean decomposition method for vibration signal. J. Data Acquis. Process..

[46] Jia L., Zhang Q., Zheng X., Yao P., He X., Wei X. (2019). The empirical optimal envelope and its application to local mean decomposition. Digit. Signal Process..

[47] Matz V., Ramos P.M., Brás N.B., Serra A.C.A. Comparative Evaluation Between Frequency Estimation Algorithms for Power Quality Assesment in DSP Implementation. Proceedings of the XVIII IMEKO World Congress, Metrology for a Sustainable Development.

